# Self-Aligned Interdigitated Transducers for Acoustofluidics

**DOI:** 10.3390/mi7120216

**Published:** 2016-11-25

**Authors:** Zhichao Ma, Adrian J. T. Teo, Say Hwa Tan, Ye Ai, Nam-Trung Nguyen

**Affiliations:** 1 Pillar of Engineering Product Development, Singapore University of Technology and Design, Singapore 487372, Singapore; zhichao_ma@mymail.sutd.edu.sg; 2 Queensland Micro and Nanotechnology Centre, Griffith University, Brisbane, QLD 4111, Australia; a.teojiantong@griffith.edu.au

**Keywords:** surface acoustic wave, acoustofluidics, microfluidics, interdigitated transducers

## Abstract

The surface acoustic wave (SAW) is effective for the manipulation of fluids and particles at microscale. The current approach of integrating interdigitated transducers (IDTs) for SAW generation into microfluidic channels involves complex and laborious microfabrication steps. These steps often require full access to clean room facilities and hours to align the transducers to the precise location. This work presents an affordable and innovative method for fabricating SAW-based microfluidic devices without the need for clean room facilities and alignment. The IDTs and microfluidic channels are fabricated using the same process and thus are precisely self-aligned in accordance with the device design. With the use of the developed fabrication approach, a few types of different SAW-based microfluidic devices have been fabricated and demonstrated for particle separation and active droplet generation.

## 1. Introduction

Acoustic waves have superior propagating properties in solids and fluids, which readily enable non-invasive and biocompatible manipulation at long operating distances. Exploiting acoustic waves for fluid and particle manipulation in microfluidics has led to the recently emerging research area of acoustofluidics [1,2,3]. Different types of acoustofluidic devices have been developed for various microfluidic applications such as particle separation [4,5,6,7], particle patterning [8,9], liquid nebulization [10], and droplet mixing and manipulation [11,12,13,14,15,16,17,18]. Compared to bulk acoustic wave-based piezoelectric transducers, surface acoustic wave (SAW)-based transducers are more preferred for integration with microfluidic systems because of their design flexibility and their ability to operate at high frequencies. The conventional approach for fabricating SAW transducers is generally to pattern metallic interdigitated transducers (IDTs) on a piezoelectric substrate. When an AC signal at a resonant frequency of the SAW is applied on the IDTs, it can generate the SAWs propagating along the substrate surface, and the shape of the SAW field is simply defined by choosing a proper design of the IDTs. The fabrication of the patterned metallic IDTs requires complex and expensive microfabrication steps, including photolithography and selective metal deposition [19]. In addition, the microfluidic channels are fabricated separately from the IDTs so that a precise and laborious manual alignment between channels and IDTs is required to form the final acoustofluidic system [20]. These complex and expensive fabrication steps crucially rely on access to clean room facilities and thus hinder the wide application of SAW-based microfluidics in biomedical research.

The ability of simple and quick development of SAW-based acoustofluidic prototypes using an affordable fabrication process, particularly without the need for clean room facilities, can greatly promote its exploratory applications in biomedical research. Rezk et al. developed an affordable and convenient solution to convert electrical signals to mechanical vibration along the piezoelectric substrate [21]. The group demonstrated that common household aluminum foil could be used to nebulize fluids and induce streaming inside a sessile droplet. However, the lamb waves generated via the aluminum foil electrodes are complex and have uncontrollable patterns, which greatly limit its wide applications compared to the patterned metallic IDTs. Previous studies show that infusing conductive materials and composites along microfluidic channels has enabled the fabrication of microscale electrodes to manipulate particles, droplets and jets [22,23,24,25,26,27,28].

In the present work, we present a simple and affordable approach to reliably fabricate SAW-based acoustofluidic devices without the need for clean room facilities. We make use of lithography to define liquid channels for sample loading and electrode channels for IDT fabrication. A metal alloy with a low melting point is infused to fully fill up the electrode channels to construct the IDTs. Since the liquid channels and the electrode channels are fabricated in the same lithography process, they are self-aligned and can be arbitrarily positioned with respect to each other in a very precise manner. We make use of this fabrication technique to develop a few types of different self-aligned SAW-based acoustofluidic devices. In particular, we demonstrated size-based particle separation and active control of droplet generation with this concept. The ability for simple and rapid prototyping of acoustofluidic devices allows researchers without microfabrication expertise and clean room access to explore acoustofluidics research, and thus broaden the impact of acoustofluidics in practical applications.

## 2. Results and Discussion

### 2.1. Design and Fabrication of Self-Aligned SAW-Based Acoustofluidic Device

**Figure 1:**
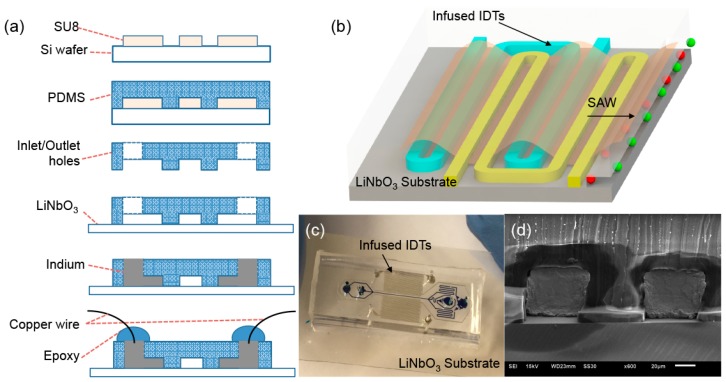
Design and fabrication of the self-aligned SAW-based microfluidic device. (**a**) Fabrication process for the self-aligned microfluidic device; (**b**) Schematic of the IDT actuation for SAW generation; (**c**) Image of one example of the self-aligned microfluidic device for particle separation; (**d**) Scanning electron microscopy (SEM) image of the device cross-section. The scale bar is 20 μm.

The self-aligned SAW-based microfluidic device comprises the infused IDTs for SAW generation and the microfluidic channels for liquid flow. The structures of the two components are defined by two types of channels, namely the liquid channel and the electrode channel, which were fabricated using the same process with a standard lithography technique as shown in Figure 1a [26,27,28,29,30,31]. Briefly, a 40-μm-thick SU-8 master mold with patterns of the liquid channel and electrode channel was first fabricated on a silicon wafer by photolithography (SU-8 25, MicroChem Corp., Newton, MA, USA) using a spin-coater and a UV flood machine within a fume hood. A degassed mixture of polydimethylsiloxane (PDMS) prepolymer and curing agent (Sylgard184 Silicone Elastomer Kit, Dow Corning Corp., Freeland, MI, USA) with a weight ratio of 10:1 was poured on the fabricated master mold and cured at 75 °C for 2 h. The cured PDMS was then peeled off and punched with 1 mm through-holes as inlet and outlet reservoirs. After an air plasma treatment for 150 s at 18 W, the surface-modified PDMS layer was bonded onto a 128° rotated Y-cut X-propagating lithium niobate wafer. All the channels were treated with Aquapel (PPG Industries, Pittsburgh, PA, USA) to render the surface hydrophobicity. A metal alloy with a low melting point (51% In, 32.5% Bi and 16.5% Sn, melting point 60 °C, Indium Corporation, Singapore) was inserted in the punched holes of the two parallel fork-shaped electrode channels and melted by placing the fabricated device on a 60 °C hotplate. The melted alloy then filled up the fork-shaped electrode channel to form the infused IDTs, and copper wires were inserted into the holes to provide external connections. Once the infused IDTs were completed, the hotplate was turned off to allow the device to cool down slowly. The melted alloy solidifies again at room temperature, forming robust and structurally stable IDTs. A drop of UV epoxy (Loctite 3526 light cure acrylic, Henkel Corporation, Rocky Hill, CT, USA) was squeezed at each wire-PDMS junction and cured after exposure to UV light to stabilize the wires.

If an AC signal is applied on the infused IDTs at their resonant frequency, the electrical energy turns into mechanical vibration waves. The waves propagate along the substrate surface through the liquid placed along the pathway (Figure 1b). The width of the electrode channels is constant and equal to the spacing between individual parallel segments of the electrode channels, similar to the conventional double-finger-type IDTs [32]. Figure 1c shows an example of the self-aligned SAW-based microfluidic devices for particle separation. Figure 1d shows the representative scanning electron microscopy (SEM) image of the cross-section of the infused IDTs.

### 2.2. Size-Based Particle Separation in a Standing SAW Field

A suspended particle exposed to a standing acoustic field is subjected to an acoustic radiation force that is dependent on its relative density and sound speed with respect to the suspension media. This force tends to attract less compressible particles (e.g., cells and polystyrene particles) to acoustic pressure nodes and more compressible particles (e.g., lipids and elastomer particles) to pressure antinodes [33,34,35]. Since this acoustic radiation force is proportional to the particle volume, size-based separation of microscale particles using standing SAWs (SSAWs) fields has been widely demonstrated [20,36,37]. In these studies, suspended particles experiencing a stronger acoustic radiation force are designed to rapidly migrate toward the pressure nodes which are located at specific locations across the microfluidic channel. Since the microfluidic channel and patterned metallic IDTs are fabricated separately, it is crucial to precisely align the channel with respect to the IDTs for keeping the acoustic pressure nodes parallel to the channel and locating the pressure nodes with a pre-designed distance (typically ~100 microns) away from the original particle positions.

Recently, an innovative design with a tilted-angle SSAW field with respect to the microfluidic channel was used for size-based separation [37,38], this eliminates the need for precisely locating the acoustic pressure nodes with specific distances away from the original particle streamline. However, an optimized angle between the channel and the IDTs is still expected, and thus a precise alignment process is still required. Our most recent study reveals that an alignment mismatch greater than 5 μm could induce strong asymmetric acoustic streaming flow patterns [7]. In contrast to these conventionally patterned IDTs with the need for a precise post-alignment process, the presented approach can easily fabricate self-aligned SAW-based acoustofluidic devices.

**Figure 2:**
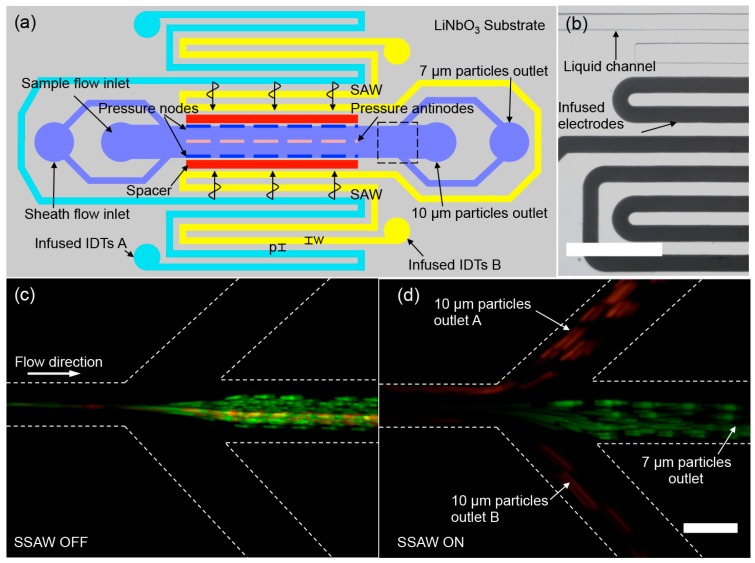
Size-based particle separation using a self-aligned, SAW-based microfluidic device. (**a**) Schematic device layout for particle separation. A sample mixture containing 7 and 10 μm particles is hydrodynamically sandwiched by two sheath flows along both sides. During the exposure to a SSAW field, the 10 μm particles with red fluorescence experiencing a stronger acoustic radiation force laterally migrate to the sidewalls of the liquid channel and flow into the two side outlets, while the 7 μm particles with green fluorescence nearly follow the original streamline into the middle outlet; (**b**) Microscopic photograph of the infused electrodes and the liquid channel. The scale bar is 500 μm. Particle trajectory at the trifurcation outlet region when the 20.0 MHz SAW is turned off (**c**) and turned on (**d**). The images of (**c**,**d**) are obtained by overlapping 100 frames recorded every 0.05 s. The scale bar is 100 μm.

We first used a self-aligned SAW-based acoustofluidic device to demonstrate size-based separation of 7 μm and 10 μm polystyrene particles in a SSAW field. Figure 2a shows the schematic design of the self-aligned SAW-based acoustofluidic device. One pair of infused IDTs was fabricated parallel along the liquid microfluidic channel. An air cavity with a width of 100 μm is placed between the infused IDTs and the liquid channel to prevent possible penetration of the electric field into the liquid channel. The liquid channel has a width of 100 μm and a height of 40 μm. The electrode channels with the same height as the liquid channel have a width of *w* = 80 μm and are uniformly spaced with *p* = 80 μm, as shown in Figure 2b. The AC signal from a RF signal generator (DSG800, Beaverton, OR, USA) amplified by a linear amplifier (Model 5303055, Ophir RF, Los Angeles, CA, USA) is applied on the two IDTs placed on both sides of the liquid channel to generate the SAWs. The self-aligned SAW-based device has multiple discrete resonant frequencies which can be determined by observing the magnitude of the acoustophoretic response of suspended particles at sweeping frequencies.

We first introduced a 10 μm polystyrene particle suspension into the liquid channel and waited until a steady-state liquid flow. Subsequently, the IDTs were excited to generate a SSAW field across the liquid channel, resulting from the constructive interference of two traveling waves propagated from both sides of the liquid channel. The frequency of the exciting AC signal was swept from 5.0 MHz to 25.0 MHz. We found that the acoustophoretic motion of particles was most responsive at 20.0 MHz. Thus, we chose *f* = 20.0 MHz as the operating frequency that corresponds to a wavelength of λ = 200 μm. The width of the liquid channel is nearly half of the wavelength, and the acoustic pressure nodes were located at the two sidewalls of the liquid channel in our device. Therefore, particles exposed in the SSAW field tend to move toward the sidewalls of the liquid channel.

In the particle separation experiments, a mixed particle suspension consisting of 7 μm and 10 μm particles was continuously introduced into the liquid channel at a flow rate of 1.0 μL/min. The two kinds of particles were hydrodynamically confined along the centerline of the channel by two faster sheath flows, both at a flow rate of 1.2 μL/min. If the IDTs were not actuated, the 7 μm (green fluorescence) and 10 μm particles (red fluorescence) both streamed into the middle outlet in the absence of the acoustic radiation effect, shown in Figure 2c. We swept the applied voltage from 0 to 50 V with a step of 2.5 V to find the optimized voltage for the separation of 7 μm and 10 μm particles. Once the IDTs were actuated at 20.0 MHz, the 10 μm particles experienced nearly three times stronger acoustic radiation force than the 7 μm particles and preferentially migrated to the two sidewalls of the liquid channel at a higher lateral velocity. Based on the observation of the separation process under the microscope, we found that the applied voltage of 42.5 V achieved 100% separation by counting 500 particles in total flowing through the field of view. As a result, with an input sinusoidal voltage of 42.5 Vpp, the larger 10 μm particles were switched to the two side outlets, while the smaller 7 μm particles remained moving to the middle outlet, as shown in Figure 2d (Video S2 in Supplementary Materials).

### 2.3. Active Control of Droplet Generation Using a Traveling SAW

**Figure 3:**
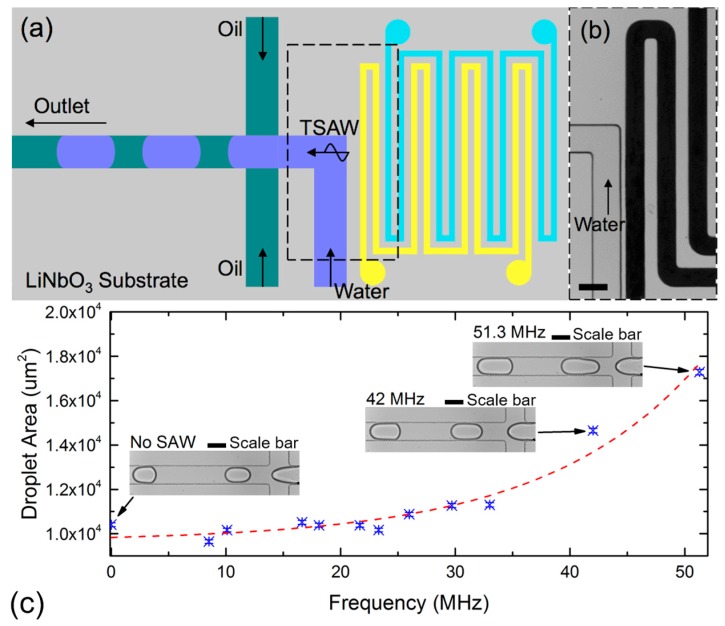
Active control of droplet generation. (**a**) Schematic sketch of the IDT and flow focusing layout; (**b**) Microscopic photograph of the infused electrodes placed at about 30 μm from the dispersed phase channel (water). The scale bar is 100 μm; (**c**) Relationship between droplet area and frequency at fixed volumetric flow rates. The dotted red line is an exponential fitting obtained from the experimental results. Images of droplets generated at selected points are shown for illustration. The scale bar is 100 μm.

In this section, we demonstrate the active control of water-in-oil droplet generation in a microfluidic flow-focusing device. The infused IDTs are placed at a distance of about 30 µm away from the dispersed phase channel. The traveling surface acoustic wave (TSAW) are generated in the direction of the dispersed flow. Figure 3a,b illustrate the schematic sketch and the image of the device configuration. Our configuration is different from the previously reported work, where acoustic streaming is induced at the continuous phase fluid [17,18]. Here, we anticipate induced acoustic streaming at the dispersed phase fluid. The induced streaming reduces the shear forces imposed by the continuous phase. As a result, the size of the produced droplets increases when the transducer is activated.

A function generator (AFG3102C, Tektronix, Beaverton, OR, USA) was used to generate the AC signal, which is conditioned by a linear amplifier (Model 5303055, Ophir RF, Los Angeles, CA, USA). A digital oscilloscope (TBS1102B, Tektronix, Beaverton, OR, USA) measured the resulting signals. The devices were mounted on an inverted microscope (Nikon Ti-E, Nikon, Tokyo, Japan) and images were captured using a high-speed camera (Miro 3, Vision Research, Wayne, NI, USA) at a frame rate of about 2000 fps. Water-in-oil droplets were generated by infusing both the dispersed and continuous phase fluids through a syringe pump (SPM 100, Singapore). The dispersed phase fluid was distilled water (DI) and the continuous phase fluid was mineral oil (M5904, Sigma-Aldrich, St. Louis, MO, USA) with 0.5% *wt*/*wt* span 80 (S6760, Sigma-Aldrich, St. Louis, MO, USA). The interfacial tension between the immiscible phases was about 6.2 mN/m. The volumetric flow rates of the dispersed and continuous phase were fixed at 50 μL/h and 200 μL/h, respectively. The size of at least 100 droplets was measured automatically using the ADM software [39]. Figure 3 depicts the relationship between the droplet area and frequency at fixed volumetric flow rates.

At fixed volumetric flow rates, and without applying the AC signal, the droplets are formed in the squeezing regime [29]. The capillary number in this case is about 0.05. This value agrees well with past reported literature [31]. The apparent droplet area is about 10,407 μm^2^. At a fixed input sinusoidal voltage of 50.8 Vpp, the droplet area increased significantly at resonance frequencies of 42.00 MHz and 51.30 MHz, respectively. Figure 3c shows the micrographs of the droplets generated at different resonance frequencies. The corresponding apparent droplet areas are observed to be increasing exponentially to 14,653 μm^2^ at 51.3 MHz (Video S3 in Supplementary Material). We hypothesize that the TSAW resulted in acoustic streaming of the fluids, which in turn increases size of the droplets formed [40]. The complex interaction between the induced streaming and droplet generation is currently beyond the scope of this communication, as our aim is to illustrate that the self-aligned transducer can be used to effectively control the size of the generated droplets.

## 3. Conclusions

We have developed a simple, affordable and innovative technique to reliably fabricate SAW-based acoustofluidic devices without the need for clean room facilities. The interdigitated transducers (IDTs) for SAW generation were constructed by infusing a low-melting-point metal alloy into channels that can be fabricated in the same lithography process of the liquid microfluidic channel. This innovative fabrication technique not only substantially reduces the cost for device fabrication, but also enables precise self-alignment of IDTs with respect to the microchannels. With this technique, liquid samples can now be precisely manipulated at arbitrary desired locations. To demonstrate the versatility of the developed fabrication technique, we fabricated self-aligned SAW-based acoustofluidic devices for size-based particle separation and active control of droplet generation. Experimental results validated the use of our unique IDTs. The technology reported here will enable simple and rapid prototyping of acoustofluidic devices, which can significantly broaden the impact of acoustofluidics in practical biomedical applications.

## Supplementary Materials

The following are available online at http://www.mdpi.com/2072-666X/7/12/216/s1, Figure S1: TSAW-induced sessile droplet streaming experiment, Figure S2: TSAW-induced sessile droplet streaming experiment at multiple resonant frequencies, Video S1: TSAW-induced sessile droplet streaming experiment, the video is recorded with a high-speed camera at frame rate of 2000 frames/s and played at 20 frames/s, Video S2: Size-based particle separation in a SSAW field, Video S3: TSAW effects in a microfluidic flow-focusing system, at 42 and 51.3 MHz, the size of the droplets produced increases at fixed volumetric flow rates, the video is played at 200 frames/s.
