# Supplementary material for: Self-Aligned Interdigitated Transducers for Acoustofluidics
Source: Micromachines (Basel). 2016 Nov 25;7(12):216. doi: 10.3390/mi7120216 (PMC6189727; doi:10.3390/mi7120216)
Supplement: Supplementary file 1 [file micromachines-07-00216-s001.zip › micromachines-162819-suppl.pdf]

Supplementary Materials: Self-Aligned Interdigitated Transducers for Acoustofluidics

Zhichao Ma, Adrian J. T. Teo, Say Hwa Tan, Ye Ai and Nam-Trung Nguyen

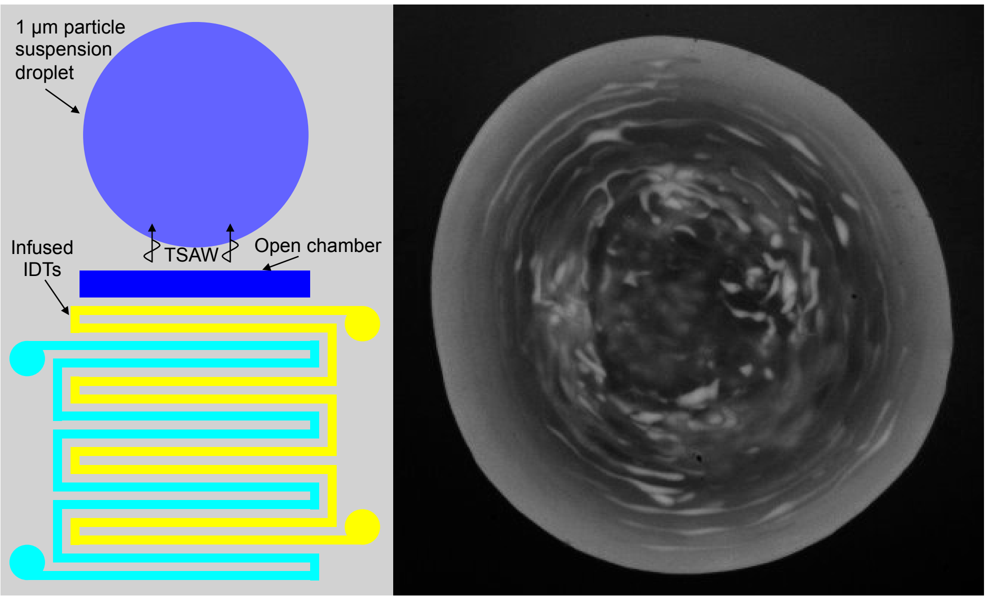

Figure S1. Traveling surface acoustic wave (TSAW)-based droplet streaming experiment. A 0.5 μL droplet with a 1 μm fluorescent particle suspension is placed in the direction of the traveling SAW generated from the infused interdigital transducers (IDTs). The uneven acoustic pressure distribution induces a streaming vortex within the droplet. Simultaneously, the incident acoustic waves interfere with the reflected acoustic waves from the droplet/air interface, thus forming a standing acoustic field within the droplet. Some of the suspended particles are aggregated to the pressure nodes in a complex pattern. The video of the particle movement and streaming flow pattern in the droplet is shown in the Supplementary video S2.

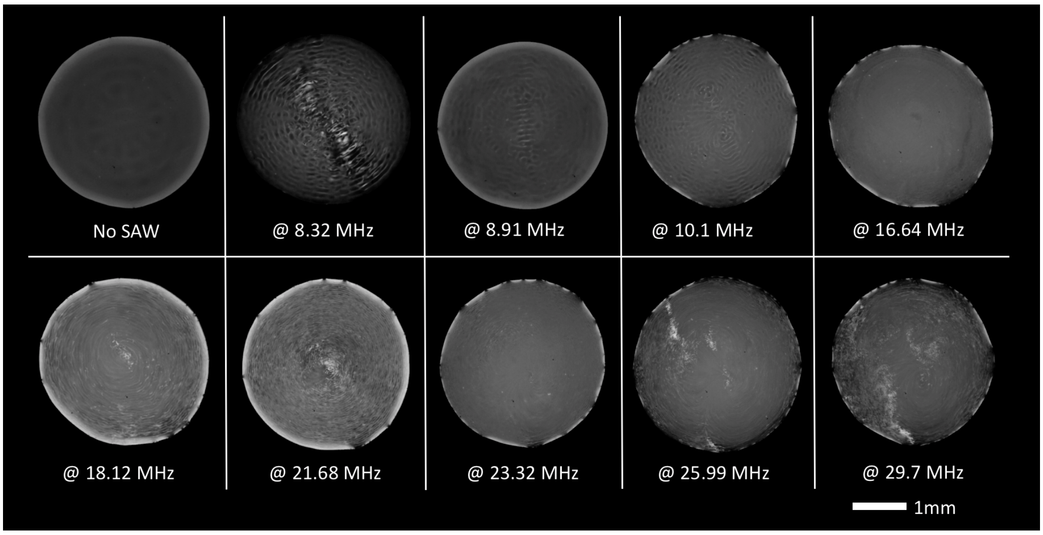

Figure S2. Droplet streaming at different resonance frequencies. The 0.5 μL droplets loaded with 0.02% volume ratio of fluorescent particles (PSF001UM, Magsphere) were pipetted and placed at about 7 mm away from the IDT. Complex standing wave formations and flow patterns were observed at different resonance frequencies. Temperature measurements at various locations show a negligible increase when the input voltage is fixed at 200 mV.
